# ASO Author Reflections: The Pursuit of Eliminating Surgery after Neoadjuvant Systemic Therapy in Breast Cancer Patients

**DOI:** 10.1245/s10434-020-09324-6

**Published:** 2020-12-10

**Authors:** Ariane A. van Loevezijn, Frederieke H. van Duijnhoven, Marie-Jeanne T. F. D. Vrancken Peeters

**Affiliations:** Department of Surgical Oncology, Netherlands Cancer Institute - Antoni van Leeuwenhoek, Plesmanlaan 121, 1066 CX Amsterdam, The Netherlands

## Past

In breast cancer patients with pathological complete response (pCR) of the breast after neoadjuvant systemic therapy (NST), the therapeutic effect of surgery is questionable. The detection of pCR without surgery, however, is challenging. Imaging techniques, including MRI and FDG-PET/CT, have not been able to reliably identify patients with pCR after NST.^1^ Therefore, several studies were initiated, aiming to identify pCR with the use of biopsies. Exploratory analyses showed promising results with false-negative rates (FNRs) of 0–5% for detecting pCR with large vacuum-assisted biopsies.^2^ However, none of these studies selected patients with radiological response using MRI, the most accurate diagnostic modality for this purpose, and sample sizes were small. In the MICRA trial,^3^ we aimed to identify pCR of the breast in patients with good MRI response using eight ultrasound-guided minimally invasive 14G core biopsies of the pre-NST marked tumor area.

## Present

Currently, the prospect of eliminating breast surgery in early-stage breast cancer patients with pCR after NST is a much-discussed topic. The results of the MICRA trial were presented simultaneously with results of three other studies aiming to identify breast pCR without surgery: a multi-institutional pooled analysis, the RESPONDER trial, and the NRG-BR005 trial.^4^,^5^ All four studies differed in biopsy technique (vacuum-assisted or core-cut, either stereotactic or ultrasound-guided), the number and size of the biopsies (14G–7G), and radiological response assessment. None of the studies achieved a FNR below 10% in the entire study population. As for the MICRA trial, the relatively small 14G biopsies mainly missed small lesions and DCIS in patients with radiological complete response on MRI.^3^

## Future

Breast cancer patients with residual disease after NST could benefit from adjuvant systemic therapy (e.g., T-DM1), and reliable assessment of residual disease is therefore essential when considering omission of surgery. The risk of sampling error in future biopsy studies may be reduced by obtaining at least six large and pathologically representative vacuum-assisted biopsies under optimal imaging conditions.^5^,^6^ In addition, adequate patient selection is important. Biomarkers such as tumor-infiltrating lymphocytes in combination with advanced MRI analysis could be used to develop a response prediction model which may even outperform biopsies in detecting pCR in selected patients in the near future. Furthermore, pCR of the breast does not guarantee pCR of the lymph nodes, which is an important prognostic factor for disease recurrence and should therefore be investigated as well.
